# Clinician-Targeted Intervention and Patient-Reported Counseling on Physical Activity

**DOI:** 10.5888/pcd11.130302

**Published:** 2014-05-29

**Authors:** Jennifer K. Carroll, Paul C. Winters, Mechelle R. Sanders, Francesca Decker, Thanh Ngo, Christopher N. Sciamanna

**Affiliations:** Author Affiliations: Paul C. Winters, Mechelle R. Sanders, Francesca Decker, Thanh Ngo, University of Rochester, Rochester, New York; Christopher N. Sciamanna, Penn State College of Medicine, Hershey, Pennsylvania.

## Abstract

**Introduction:**

Limited time and lack of knowledge are barriers to physical activity counseling in primary care. The objective of this study was to examine the effectiveness of a clinician-targeted intervention that used the 5As (Ask, Advise, Agree, Assist, Arrange) approach to physical activity counseling in a medically underserved patient population.

**Methods:**

Family medicine clinicians at 2 community health centers were randomized to Group 1 or Group 2 intervention. Both clinician groups participated in 4 training sessions on the 5As for physical activity counseling; Group 2 training took place 8 months after Group 1 training. Both groups were trained to refer patients to a community exercise program. We used a pre–post analysis to evaluate the effectiveness of the intervention on clinician use of 5As. Eligible patients (n = 319) rated their clinicians’ counseling skills by using a modified Physical Activity Exit Interview (PAEI) survey. Clinicians (n = 10) self-assessed their use of the 5As through a survey and interviews.

**Results:**

Both patient and clinician groups had similar sociodemographic characteristics. The PAEI score for both groups combined increased from 6.9 to 8.6 (on a scale of 0–15) from baseline to immediately postintervention (*P* = .01) and was 8.2 (*P* = .09) at 6-month follow-up; most of the improvement in PAEI score was due to increased use of 5As skills by Group 2 clinicians. Group 1 reported difficulty with problem solving, whereas Group 2 reported ease of referral to the community exercise program.

**Conclusion:**

A clinician training intervention showed mixed results for 5As physical activity counseling.

## Introduction

Americans made 560 million visits to primary care physicians in 2010 (1). Most visits were for prevention and treatment of chronic conditions for which physical activity counseling would be appropriate. Even modestly effective evidence-based physical activity interventions in primary care settings could have powerful health benefits for patients (2).

Yet clinicians face significant barriers to implementing evidence-based interventions, especially for underserved populations that have limited resources and competing demands (3). The implications of clinicians not using evidence-based interventions include worsening health disparities related to inadequate physical activity.

Some barriers to implementing evidence-based interventions could be addressed by the “5As,” an evidence-based clinical counseling framework in which clinicians are encouraged to Ask about (or Assess), Advise on, Agree upon, Assist with, and Arrange follow-up on patients’ behavior-change efforts (4–11). The 5As can be used briefly and during multiple visits (12) and have been endorsed by the US Preventive Services Task Force (13) and others (14–16) and more recently for obesity counseling (17,18).

Despite recommendations to use the 5As, evidence for the feasibility of implementing the 5As in “real-world” settings is limited. In 2010, the US Preventive Services Task Force updated its recommendations on physical activity counseling (19), concluding that the evidence for low-intensity (30 minutes or less) counseling interventions was mixed and that further study is needed to evaluate them. Low-intensity counseling interventions are especially important because they are more likely than high-intensity interventions to be implemented in primary care settings.

The objective of this study was to examine the effectiveness of a clinician-targeted intervention that used the 5As approach to physical activity counseling in a medically underserved patient population. 

## Methods

The study design and protocol are described in detail elsewhere (20). Briefly, the study used a 2-group pragmatic pilot randomized-controlled–trial design; 13 clinicians were randomized to receive the intervention; patients (n = 319) were the primary unit of analysis. For practical and logistical reasons, the clinicians were randomized so that Group 1 clinicians (n = 6) participated in the intervention first; Group 2 (n = 7) participated in the intervention 8 months later. The intervention consisted of a clinician training program designed to increase use of the 5As for physical activity counseling with patients. Outcomes were patient and clinician assessments of the effectiveness of the intervention in increasing use of the 5As for physical activity counseling. Other than the timing, there was no difference in the intervention or the assessment for the 2 groups. The University of Rochester Review Board approved the study protocol. The intervention period started in June 2009 and ended with the final follow-up data collection in October 2011.

### Recruitment, enrollment, randomization of clinicians and patients

Clinicians and patients were recruited through 2 federally qualified health centers in Rochester, New York, serving a predominantly low-income, racially/ethnically diverse population of 14,000 patients. Details on inclusion and exclusion criteria for participants are described elsewhere (20). Clinicians were eligible if they practiced at one of the aforementioned centers. Clinicians were recruited, enrolled, and randomized to Group 1 or 2 from January through March 2009 before patient recruitment began (20). Of 15 clinicians eligible, 13 consented to participate (9 family physicians, 2 family nurse practitioners, and 2 physician assistants) and were randomized. Of the 13 clinicians randomized, 2 clinicians relocated and one retired, leaving 10 for analysis (5 in Group 1 and 5 in Group 2). Neither clinicians nor research staff was blinded to the assignment of clinicians. Clinicians were paid up to $365 for participation in all study activities.

Patients were eligible if they were currently enrolled at one of the health centers, were aged 18 years or older, were able to provide informed consent, and made a health maintenance visit or follow-up visit for a chronic condition for which physical activity counseling would be appropriate. Nurse assistants mentioned the study to the patients when they were in the examination room. If the patient expressed interest, a research assistant entered the room to provide additional details about the study; patients who agreed to participate signed an informed consent form. For ethical and regulatory reasons, patients were not blinded to study topic and objective; as part of the informed consent process, they were made aware of these. However, they were blinded to time point (baseline, postintervention, 6-month follow-up) and did not know whether or when their clinicians took part in the intervention; patient participants were paid $20 for participation.

During the study period, 1,029 patient participants were assessed for eligibility. Of these, 481 (or 46.7% of those assessed) were ineligible. Among the remaining 548 patients, 325 consented to participate (Figure). Of the 325 patients, 319 completed surveys for analysis. 

**Figure:**
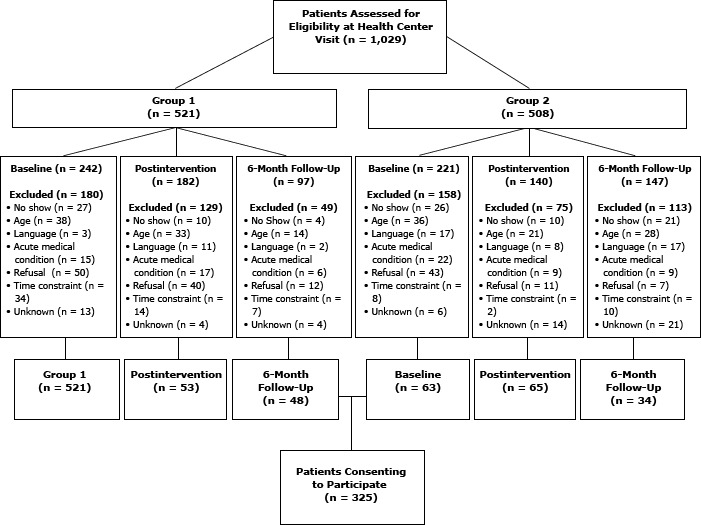
Assessment of patient participant eligibility and reasons for exclusions from study. Within each group, patients at baseline, postintervention, and 6-month follow-up were assessed independently of one another (ie, patients participating at baseline did not necessarily participate at postintervention or 6-months postintervention).

### Intervention

The intervention consisted of 4 one-hour training sessions for clinicians at the health center sites (20). Clinicians were trained through techniques known to be effective for communication training (21–23): didactic materials, skills and competency checklists, role-play, and cognitive rehearsal. Training topics consisted of introduction to the 5As, current recommended guidelines for physical activity, techniques to elicit patient motivation, strategies to problem-solve, tips on documentation in electronic health records, and a list of community resources for physical activity. As part of the Assist and Arrange steps, clinicians were taught to refer patients to a community exercise program (the Healthy Living Program), which had partnered with the federally qualified health center. The training period was interactive, and at its conclusion, each clinician received a competency checklist noting their accomplishment of the 5As completed by a standardized patient (a person who realistically portrays a patient with a certain health condition). No booster sessions or other follow-up training activities were provided. All training sessions were audio-recorded. Two students listened to the recordings and completed a checklist of predefined training goals and activities to ensure fidelity.

### Patient-reported outcome measures

For each clinician, patients completed a survey at 3 points: baseline, immediately postintervention, and at 6-month follow-up. Each patient completed 1 survey, so different groups of patients provided surveys at each point. Patients completed the survey, a modified version of the Physical Activity Exit Interview (PAEI) (24), after their office visit. The PAEI is a validated 12-item measure asking patients to report clinician use of 5As for physical activity counseling. Patients dichotomously (yes/no) answered questions such as, “Did your doctor advise you to become more physically active?”; “Did your doctor discuss difficult situations you might encounter or problems you might have in trying to become more physically active?”; and “Did you and your doctor put the plan to become more physically active in writing?” The PAEI was modified for this intervention by adding 3 questions: “Has your provider ever asked you about your confidence to change your exercise habits?”; “Has your provider ever asked you about your willingness to change your exercise habits?”; and “Has your provider ever referred you to other programs, resources, consultants, etc., to help you with physical activity?” The score range for the modified PAEI was 0 to 15 (ie, 1 point for each yes). The patient survey also asked about sociodemographic characteristics, barriers to physical activity, and support and resources for physical activity.

### Clinician baseline and follow-up assessments

Each clinician completed baseline and 6-month follow-up surveys. The surveys asked about demographic characteristics, current practice of physical activity counseling, confidence in using 5As in counseling on physical activity, and knowledge of resources that could meet their patients’ needs. Their confidence in using counseling skills was self-rated on a 5-point Likert scale (1 = not confident, 5 = very confident). At follow-up, each clinician also completed a 30-minute interview to provide feedback about the overall experience in the intervention, changes in skills, problems or difficulties, training techniques, satisfaction with the intervention, and suggestions for improvement.

### Statistical analysis

All analyses were based on a significance level of .05. Two-sample *t* tests for continuous variables and χ^2^ tests for categorical variables were used to evaluate the success of the randomization in balancing baseline covariates between groups. From the power and sample-size analysis, given our sample size, we could detect a difference of 1.5 or greater in PAEI scores between baseline and follow-up with 80% power. Data were analyzed with generalized estimating equations (GEE) models with patients nested within clinician using a negative-binomial link to account for the distribution of PAEI scores. We chose to use GEE models to account for nesting of patients within clinician and a possible unknown correlation between outcomes and to obtain robust standard errors. Also, GEE models provide a practical method for analyzing conditional responses that may depart from the normality assumption required for general linear models. Finally, we determined effect sizes and categorized them as small, medium, or large (25). For the qualitative analysis of interviews, we conducted interviews of patients and clinicians after the patient visit. All interviews were audio-recorded, transcribed, coded, and analyzed by using a constant comparative analysis technique.

## Results

Three-quarters of clinicians were women; 66% were white, 25% African American, and 9% Asian. The average age of clinicians was 49 years (range, 31–73 y), and the average length of practice was 15 years (range, 2–33 y). Group 1 and Group 2 patients did not differ significantly by any sociodemographic or health characteristic, so data for both groups were combined (Table 1). Patients’ mean age was 43 years, and 69.9% were African American. Most (61.4%) had public insurance, and the mean body mass index (BMI) was 32.3. The most common weight-related conditions were hypertension (48.5%), chronic pain (42.3%), depression (32.4%), and diabetes (21.3%); 70% of patients had more than 1 weight-related chronic condition, and 51% had 3 or more conditions.

**Table 1:** Sociodemographic and Health Characteristics of Patient Participants (N = 325), Study on Clinician Counseling on Physical Activity, 2009–2011^a^

Characteristic	Value
**Sex**	
Female	225 (70.5 )
Male	94 (29.5)
**Race/ethnicity**	
Hispanic	28 (8.8)
Non-Hispanic white	53 (16.6)
Non-Hispanic black	223 (69.9)
Other	15 (4.7)
**Insurance**	
Public	156 (61.4)
Private	96 (37.8)
None	2 (0.8)
**Education**	
Less than high school	86 (27.7)
High school or equivalent	100 (32.3)
More than high school	124 (40.0)
**Employment**	
Employed	112 (34.8)
Not employed	210 (65.2)
**Annual income, $**	
<10,000	119 (39.8)
10,000–20,000	90 (30.1)
>20,000	49 (16.4)
Chose not to answer	41 (13.7)
**Age, mean (SD), y**	43.2 (14.2)
**BMI, mean (SD), kg/m^2^**	32.3 (8.7)
**Chronic condition**	
Obesity (BMI ≥30)	174 (58.2)
Hypertension	157 (48.5)
Chronic pain	137 (42.3)
Depression	105 (32.4)
Diabetes	69 (21.3)
High cholesterol	78 (24.1)
≥3 Chronic conditions	164 (51.3)

Abbreviations: SD, standard deviation; BMI, body mass index.

a Values are numbers and percentages, unless otherwise indicated. Because of missing data, some categories do not sum to 325. Percentages are based on number of participants who responded to a particular question.

In the mixed model controlling for clinician as a random effect, the PAEI score for both groups combined increased from 6.9 at baseline to 8.6 at postintervention (*P* = .01) (Table 2). The medium effect size (0.34) was not sustained at 6-month follow-up (mean PAEI score at 6 months, 8.2; *P* = .09). The average PAEI score did not change significantly at either point for Group 1, whereas the average PAEI score increased at both points (from baseline) for Group 2 (Table 2). The PAEI score at baseline for Group 1 (7.5) was higher than the score at baseline for Group 2 (6.3) because Group 1 patients rated their clinicians higher on Assess and Assist items. However, at 6-month follow-up, Group 2 patients rated their clinicians higher on all PAEI items than did Group 1 patients, especially for Assess and Assist (Table 3). Of the 3 PAEI items added for this intervention, the scores for 2 items increased (Table 3).

**Table 2:** Overall Physical Activity Exit Interview (PAEI) Scores **a** at Baseline, Postintervention,^b^ and 6-Month Follow-Up, Study on Clinician Counseling on Physical Activity, 2009–2011

Value	Group 1 (n = 163)			Group 2 (n = 162 )			Both Groups (n = 319)		
	Baseline	Post	6 Months	Baseline	Post	6 Months	Baseline	Post	6 Months
**PAEI score (*P* value)^c^**	7.5 (—)	8.5 (.27)	7.5 (.96)	6.3 (—)	8.6 (.01)	9.1 (.006)	6.9 (—)	8.6 (.01)	8.2 (.09)
**Effect size^d^**	—	0.21 (Small)	0.49 (Medium)	—	0.46 (Medium)	0.46 (Medium)	—	0.34 (Medium)	0.42 (Medium)

Abbreviations: —, does not apply.

a The 12-item PAEI (24) was modified for this intervention by adding 3 questions; each question was answered by yes or no, and each yes was counted as 1 point for a possible score range of 0 to 15 points. The survey was administered to 2 groups (Group 1 and Group 2) of patients who were asked to rate their physicians (n = 10) on their physical activity counseling.

b Immediately postintervention.

c *P* values (*F* test) determined by comparing postintervention and 6-month follow-up scores with baseline.

d Effect sizes are categorized as small (0.2), medium (0.5), or large (0.8) (25).

**Table 3:** Physical Activity Exit Interview (PAEI) Scores **a** for Each of the 15 Survey Items at Baseline, Postintervention,^b^ and 6-Month Follow-Up, Study on Clinician Counseling on Physical Activity, 2009–2011

5A Question	Group 1 (n = 163 ), %			Group 2 (n = 162 ), %		
	Baseline	Post	6 Months	Baseline	Post	6 Months
**Ask**						
Has your provider ever discussed your physical activity?	84.8	96.2	83.3	81.7	81.5	87.5
**Advise**						
Has your provider ever advised you to become more physically active?	64.4	78.4	70.8	60.0	70.8	83.9
Has your provider ever discussed the reasons you might have to want to become more physically active?	59.3	72.6	64.6	63.3	70.8	74.2
**Assess**						
Has your provider ever discussed your past experiences with physical activity?	53.3	66.0	57.5	55.0	61.5	68.8
Has your provider ever discussed difficult situations you might encounter or problems you might have in trying to become more physically active?	55.9	44.9	46.8	44.1	54.7	62.5
Has your provider ever asked you about your willingness to change your exercise habits?^c^	51.7	58.8	54.2	45.0	63.1	65.6
Has your provider ever asked you about your confidence to change your exercise habits?^c^	47.5	52.0	48.9	38.6	59.4	59.4
**Assist**						
Has your provider ever discussed how frequently you should exercise?	61.7	62.0	62.5	46.7	67.7	75.0
Has your provider ever discussed how long you should exercise?	50.0	49.0	51.1	35.6	66.2	62.5
Has your provider ever discussed how hard you should exercise?	38.3	35.3	37.5	25.0	59.4	56.3
Has your provider ever discussed the types of exercise you should do?	55.0	64.7	56.3	45.9	58.5	62.5
Have you and your provider ever put the plan to become more physically active in writing?	18.3	19.6	20.8	10.2	21.5	20.6
Has your provider given you any written materials about physical activity or exercise during a clinic visit?	39.3	35.3	31.3	22.0	33.9	36.4
**Arrange**						
Has your provider said that he/she is planning to discuss your physical activity on a future visit?	38.3	56.9	37.5	36.2	49.2	54.6
Has your provider ever referred you to other programs, resources, consultants, etc., to help you with physical activity?^c^	38.3	47.1	41.7	39.7	47.7	40.6

a Scores were converted to percentage of patients answering yes to a given PAEI item. The 12-item PAEI (24) was modified for this intervention by adding 3 questions; each question was answered by yes or no, and each yes was counted as 1 point for a possible score range of 0 to 15 points. The survey was administered to 2 groups (Group 1 and Group 2) of patients who were asked to rate their physicians (n = 10) on their physical activity counseling. Generalized estimating equation models controlled for nesting of patients within clinician.

b Immediately postintervention.

c Question added to PAEI for this intervention.

From baseline to 6-month follow-up, clinician confidence increased significantly for assessing an exercise history, negotiating an exercise plan, turning setbacks into learning, helping patients cope with barriers, counseling in a cost-effective way, and having knowledge of community resources that could meet patients’ needs (Table 4).

**Table 4:** Self-Assessed Confidence^a^ of Clinicians (n = 10) in Ability to Counsel Patients on Physical Activity at Baseline and 6-Month Follow-Up, Study on Clinician Counseling on Physical Activity, 2009–2011

Counseling skill	Baseline, Mean (SD)	Follow-up, Mean (SD)	*P* Value^b^
Assess exercise history	3.1 (1.0)	4.3 (0.6)	.003
Adapt counseling to patient situation or needs	2.8 (0.7)	3.8 (1.3)	.06
Negotiate an exercise plan	2.8 (0.8)	4.0 (0.9)	.02
Turn setbacks into learning	2.2 (0.7)	3.4 (1.7)	.04
Help cope with barriers	2.4 (0.9)	4.1 (1.9)	.02
Counsel in cost effective way	2.0 (0.7)	3.5 (1.4)	<.001
Knowledge of resources that could meet your patients' needs	2.1 (0.8)	3.5 (1.2)	.004
Integrate counseling into visit	3.2 (0.7)	4.0 (1.1)	.04

Abbreviations: SD, standard deviation.

a Based on Likert scale ranging from 1 (not at all confident) to 5 (very confident).

b Paired *t* test.

### Qualitative feedback from clinicians

From the clinician interviews, some differences emerged for Groups 1 and 2 in their perceptions of their 5As counseling skills. Group 1 clinicians (whose score did not change) commented frequently on the challenges of problem solving with patients (“getting lost in the barriers” as stated by 1 clinician). Group 1 clinicians felt that the training was too brief and that booster trainings would have been helpful. They requested more feedback on their counseling skills (either from the research team or their peers) and support beyond the office visit to encourage patients and help with problem solving. In contrast, Group 2 clinicians (whose PAEI score was lower than Group 1’s at baseline but increased at follow-up) mentioned the value and ease of referral to the community exercise program. The partnership for referral to the community exercise program was newly initiated when Group 1 took part in the study and was more established by the time Group 2 took part. Group 2 clinicians also commented that the 5As framework was useful for raising awareness and reminding them of the importance of counseling on physical activity.

## Discussion

This study evaluated the effect of a clinician-targeted intervention on patient- and clinician-reported use of the 5As for physical activity counseling. The intervention used innovative, interactive clinician training techniques and focused exclusively on a medically underserved population not well represented in this type of research. The main finding was that the intervention increased patient-reported improvements in 5As counseling on physical activity immediately postintervention but not at 6-month follow-up, although this improvement was primarily due to higher scores for Group 2 clinicians. Group 1 clinicians had a higher baseline PAEI score, which may have limited the effectiveness of the intervention for them. Also, the community exercise program may have provided a greater incentive for Group 2 clinicians to use the 5As because the program was better established for them than for Group l clinicians.

There are some practical lessons learned from this study. First, the development of the referral process for the community exercise program took time to function effectively, and this delay affected clinician behavior (ie, familiarity with and ease of referral to the program). Research on the effectiveness of community exercise programs for clinician referrals is lacking; others have commented on the potential demand and the difficulty of establishing effective partnerships for referral. A recent meta-analysis of primary care–based physical activity counseling (26) and recent US Preventive Services Task Force recommendations (27) identified evaluation of referral programs as a major gap in knowledge that requires future study.

Second, the intervention might have been more effective in increasing clinicians’ use of the 5As if it had included strategies such as booster trainings, reminders or prompts about the ongoing availability of the community exercise program, and additional staff support to help with problem solving (28). Consistent with their patients’ aggregate ratings, Group 1 clinicians reported challenges in problem solving with their patients; problem solving is necessary to accomplish Assess and Assist and may be related to a clinician quality that needs further study.

This study has several limitations. Although clinicians were randomized for practical reasons, we had inadequate control group data and therefore used a pre–post analysis. Although the patient sample size was larger than is typically reported for this type of research, patients were nested within a small sample of clinicians from a single geographic location. Participants’ awareness of the study topic and objective could have increased the likelihood of patient bias to overreport physical activity counseling. However, the blinding of patients to time point and participation of clinicians in the intervention increased the likelihood that any bias was evenly distributed among time points and clinicians. Finally, by design, this study did not assess patients’ behavior change during the study; instead, it aggregated patient ratings nested by clinician. This trade-off was made for practical and logistical reasons because of the focus on an underserved population, but future research would be enhanced by longitudinal assessments of changes in physical activity among patients.

Strengths of the study are that, to our knowledge, this is the first study to assess the effectiveness of a clinician intervention on use of the 5As for changing physical activity counseling in a medically underserved population. This study is relevant for several reasons. First, it addressed the high prevalence of lifestyle-related chronic conditions in a medically underserved population and the need to eliminate racial/ethnic disparities (20). Second, the intervention was interactive and innovative and had multiple levels. Although it focused on clinicians, it also created tools in the electronic health record for use by the entire clinic, and it initiated a partnership with a community exercise program. Third, this study represents an evaluation of an intervention aimed at translating guidelines into everyday practice, which is not commonly reported in the literature.

This study has 2 main clinical implications. First, clinicians can be taught to improve their physical activity counseling by using the 5As framework. Second, the intervention can improve confidence in counseling skills among physicians, especially by educating them about community resources for physical activity.

This study also has policy relevance. Primary care is undergoing transformation in the United States. The patient-centered medical home (PCMH) initiative has created a resurgence of interest in helping primary care patients change health behaviors (29) to qualify for higher insurance reimbursements (30). The PCMH standards from the National Committee for Quality Assurance include key (“must-pass”) elements for practices to “support self-care” in part by providing educational resources, self-management tools, and counseling to adopt healthy behaviors for least 50% of patients (31). The changes primary care practices are undertaking are complex, however, and many practices struggle with how to best meet the PCMH standards. This study represents 1 strategy to offer training and tools to help clinicians translate evidence-based guidelines into practice and to address the PCMH-relevant goal of helping patients adopt healthy behaviors.

The results of a clinician-directed intervention designed to increase patient reports of physical activity counseling were mixed. Group 2 clinicians (who took part in the intervention after Group 1) increased their use of the 5As more than did Group 1; the difference in use between the 2 groups was due primarily to greater use of Assess and Assist skills among Group 2. Group 2 clinicians improved their awareness of the community exercise program for referral, whereas Group 1 clinicians reported difficulty with problem-solving skills. Future directions are to explore the association between 5As counseling and patient outcomes such as enrollment and participation in community exercise programs.
